# Family History of Diabetes: Neighborhood and Familial Risks in African American Youth Living in Public Housing

**DOI:** 10.3390/healthcare13172098

**Published:** 2025-08-23

**Authors:** Ngozi V. Enelamah, Andrew Foell, Melissa L. Villodas, Chrisann Newransky, Margaret Lombe, Von Nebbitt, Mansoo Yu

**Affiliations:** 1 Department of Social Work, University of New Hampshire, Durham, NH 03824, USA; ngozi.enelamah@unh.edu; 2 Jane Addams College of Social Work, University of Illinois Chicago, Chicago, IL 60607, USA; afoell@uic.edu; 3 Department of Social Work, George Mason University, Fairfax, VA 22030, USA; mvilloda@gmu.edu; 4 School of Social Work, Adelphi University, Garden City, NY 11530, USA; cnewransky@adelphi.edu; 5 School of Social Work, Boston University, Boston, MA 02215, USA; lombem@bu.edu; 6 School of Social Work, Morgan State University, Baltimore, MD 21251, USA; von.nebbitt@morgan.edu; 7 School of Social Work, College of Health Sciences, University of Missouri, Columbia, MO 65211, USA; 8 Department of Public Health, College of Health Sciences, University of Missouri, Columbia, MO 65211, USA

**Keywords:** African Americans, adolescents, public housing, diabetes, substance-related disorders, family health, minority families

## Abstract

**Background/Objectives**: Recent data shows increasing diabetes prevalence among African Americans. Youth with a family history of diabetes are at high risk for diabetes. This study explores the multilevel risk factors associated with a family history of diabetes among African American youth in public housing. **Methods**: This study used a cross-sectional, quantitative, and community-based participatory research (CBPR) approach. The research team, comprising community stakeholders and academic researchers, employed respondent-driven sampling (RDS) for data collection (survey) and used univariate and bivariate analyses to examine variable relationships. A sequential logistic regression highlighted factors influencing the likelihood of having a family history of diabetes. **Results**: The final sample (n = 190, mean age 18.5 years, 58% female) included 35% of youth with a family history of diabetes. Forty-six percent reported medium to severe household hardships. Results suggest that reporting a family history of diabetes is correlated with maternal substance use (tau-b = 0.27 **) and alcohol problems (tau-b = 0.16 ***), paternal substance use (tau-b = 0.17 *), and eating fewer fruits (tau-b = 0.17 *). With an odds ratio (OR) of 1.70 [0.68, 4.13] and attributable fraction among the exposed at 41.3%, the final model (3) was not significant [χ^2^ = 11.19(8)]. Thus, we fail to reject the null hypothesis that the model fits the data well. Fewer vegetable consumption (OR = 15.08, *p* < 0.001), higher soda consumption (OR = 0.06, *p* < 0.001), severe household hardships (OR = 5.82, *p* < 0.01), and maternal substance use problems (OR = 6.81, *p* < 0.05) predicted a higher likelihood of a history of diabetes. **Conclusions**: Our study calls attention to the need to reevaluate interventions for hardships and substance use in diabetes management, particularly in poor neighborhoods and among minority families.

## 1. Introduction

Approximately 1.2 million Americans are diagnosed with diabetes annually [1]. Indeed, diabetes is a global public health concern [2]. A crude estimate of 38.1 million adults aged 18 years and above (14.7% of all U.S. adults), up from 34.1 million in 2020, have diabetes, including 8.7 million (3.4%) individuals 18 years and older who were unaware or had undiagnosed diabetes [1,3]. These figures exceed the U.S. projected increase to 29 million by 2050 [4,5]. Furthermore, one in four people with diabetes and one in three people with prediabetes are unaware of their condition [6,7,8]. In addition to being the seventh leading cause of death in the U.S. [9], the annual costs of diagnosed and undiagnosed diabetes exceeded $400 billion in 2022 [10,11]. Diabetes disparities highlight patterns where African Americans are 73% more likely to have both diagnosed and undiagnosed diabetes than their White counterparts [9,12]. African Americans are also more likely to receive lower-quality care, resulting in a higher burden of complications such as retinopathy, limb amputation, stroke, and cardiovascular disease from uncontrolled diabetes [13,14]. Particularly vulnerable are African American youth, who have an increased likelihood of diagnosis, twice that of non-Hispanic White individuals [12,15]. Between the ages of 10 and 19 years, non-Hispanic Black children and adolescents experienced the highest incidence of Type 2 diabetes. Factors such as racism, low educational attainment, poverty, neighborhood conditions, and housing quality issues increase the susceptibility of African American families to diabetes [9]. In addition, a family history of diabetes, a significant indicator of risk [16], has been linked to the likelihood of an individual experiencing a diabetic condition [17]. However, not a lot is known about the mechanisms associated with family histories of diabetes among African American youth, particularly those who grow up in severely distressed urban neighborhoods that increase risks for health disparities. We address this gap in knowledge using data from a sample of African American youth living in public housing to explore the multilevel risk factors associated with a family history of diabetes.

### Review of the Literature

Diabetes among African Americans. African Americans have a disproportionate burden of diabetes and diabetes risk [7,18]. Over 13% of African Americans report a diabetes diagnosis compared with 5.6% of Whites [7,14,18]. Among Americans aged 10 to 19 years, higher rates of new cases of both type 1 and type 2 diabetes were seen in African American youth compared to all other racial-ethnic groups [19]. Furthermore, once diagnosed, African Americans tend to have worse health outcomes than other groups, including higher risks of mortality from diabetes [20,21]. African American youth face additional adverse outcomes from diabetes that range from chronic stress, reduced productivity at school or work, and material deprivation from the financial cost of managing a diabetic condition for themselves or family members [15].

Disparities in diabetes incidence and prevalence, along with diabetes risks, in part result from differences in the social and physical environments occupied by African American families. Evidence points to a race-poverty-place gradient for diabetes prevalence in African Americans [17,22]. African Americans are more likely to live in poor urban neighborhoods characterized by limited resources that support physical activity and access to healthy nutrition compared to other racial groups [23]. These neighborhoods are also associated with risk factors, including food deserts, lower likelihood of having recreational facilities, and poor-quality housing [24,25]. These factors, collectively categorized as social determinants of health (SDoH), include housing conditions and access to safe and healthy neighborhoods. Thus, multiple factors in the physical and social environments impact family health history (FHH), including the incidence and prevalence of diabetes among African Americans.

Family health history and youth health outcomes. Research connects social and family health history (FHH) to increased risk for diabetes, especially in African Americans [7,16]. Despite this, FHH continues to be underutilized in the prevention and care of young people in low-resource communities because individuals in this group often lack knowledge about their FHH and have a limited understanding of the benefits of an FHH-based risk assessment [16]. For African American youth, there may be an added barrier of limited family health communication, not unrelated to the mistrust of the healthcare system, where minority families are not often open to sharing a family member’s health information with their providers. Given that family members share not only their genes but also their environment, lifestyles, and habits, there is a need to understand how these factors may influence the risk for diabetes, thus reducing risks and guiding preventive interventions.

Household and family factors—hardship. Given the intricate and resource-dependent requirements for nutrition to manage diabetic conditions, studies show that socioeconomic status and experience of hardship influence the risk of disease and are risk factors for glycemic control and complication development in diabetes [17]. Further, there is an increased risk for retinopathy, compromised kidney function, and foot infections related to insulin that are more predominant among the African American population [13,14]. This may be related to poor living and housing conditions, and lead to complications from infection. Additionally, household hardships may interfere with diabetes treatment by limiting the resources needed to follow evidence-based care recommendations, including medication access and adherence. Household hardships may also impact access to high-quality preventive care, a critical strategy for early disease detection and management [26].

Household and family factors—Parental substance use. Parental substance abuse disrupts processes in children starting from the fetus. Smoking, chronic heavy alcohol consumption, and other forms of substance use predispose children to diabetes and other forms of metabolic dysfunction [27]. More so, adolescents with diabetes were more likely to use substances and experience higher prevalence and incidence of diabetes [20,28]. Overall, previous studies found that people with behavioral health disorders, including substance use problems, made up 82% of diabetic patients [29]. Further, the incidence of diabetes is higher for children who have experienced at least one form of adverse childhood experience (ACE), including parental substance use [30,31].

Neighborhood factors—food accessibility. Neighborhood conditions influence diabetes risk through various pathways, including the unavailability of healthy foods. For instance, the concept of a “food desert” emerged to assess and highlight the accessibility and availability of fresh, healthy, and affordable food [25]. Typically, low-income and minority neighborhoods with limited access to staple foods are classified as food deserts, compared to affluent White neighborhoods, due to their limited access to supermarkets or large grocery stores [32]. Poor minority neighborhoods also suffer from greater availability of unhealthy food options, such as fast food and soda. Indeed, the general trend is that low-income, minority residents have poor access to supermarkets and healthy foods while facing greater access to fast food outlets and energy-dense foods [33,34]. This, coupled with the distance to supermarkets, limited walkability, is a factor that is positively associated with physical activity and negatively related to the prevalence of obesity, a risk factor for diabetes [35,36].

Theoretical Framework: Social determinants of health (SDoH). Using the SDoH framework, this study posits that equity in outcomes for chronic health conditions such as diabetes is influenced by individual, social, and structural factors [37]. We explored the relationship between individual, parental, household, and community-level factors that are linked to a family history of diabetes. We posit that intermediary determinants of health, reflected in individual and social level factors, including housing and neighborhood conditions, contribute to potential differences in exposure and vulnerability to environments that compromise individual health outcomes [37]. The structural level factors operate through a series of determinants at both the individual and social levels that influence health outcomes, including diabetes, and factors such as social support and access to healthcare [37,38]. Furthermore, several social determinants of having a family history of diabetes have not been explored among African American youth. Thus, this study investigated the multilevel risk factors associated with a family history of diabetes among African American youth in public housing using the following questions:

- What is the prevalence of a family history of diabetes among African American youth living in public housing?
- To what extent are (a) parental, (b) household, and (c) neighborhood factors correlated with having a family history of diabetes?
- How do (a) parental, (b) household, and (c) neighborhood factors influence the likelihood of having a family history of diabetes?

## 2. Materials and Methods

Study design and research setting. Using a community-based participatory research (CBPR) approach, which incorporated a cross-sectional design and quantitative methods, the research team (i.e., community stakeholders and academic researchers that constituted a community advisory board, CAB) recruited and subsequently collected data from African American adolescents and emerging adults residing in an extensive public housing development in West Baltimore, MD. Community stakeholders included residents of the public housing development and a social service agency located within the development. Academic researchers worked collaboratively with community stakeholders to identify salient issues and challenges, particularly those affecting youth, emerging adults, and their families.

Sampling and recruitment. The research team determined that Respondent-Driven Sampling (RDS), a form of chain referral sampling, would be the most appropriate strategy given the tight social ties among the youth and emerging adults. As a method, RDS is used for conducting research in highly stigmatized populations, where distrust is often prevalent, and individuals have strong privacy concerns [39]. The RDS process involved recruiting an initial group of youth, referred to as index cases, then systematically identifying participants who emanated from the index cases. This sampling technique corrects sampling biases typically associated with chain-referral procedures and produces a sample that is independent of the index cases. The research team posted flyers that highlighted a brief overview of the study, the date and location for data collection, contact information for key research personnel, and the IRB office at Washington University in St. Louis (Approval Number EO4-65) in the community center and surrounding agencies near the public housing development. For this study, twenty-five index youth were recruited. In compliance with the RDS, each index case was allowed to make only three referrals. Youth, 17 years or younger, were required to provide signed parental consent and signed youth assent. Participants aged 18 or older were required to provide a signed consent form. All consent forms were secured on the day the data were collected. Respondents met in groups of 5 to complete the self-administered surveys, which took 30–45 min. Youth members of the research team emphasized the need for privacy; therefore, the survey was conducted in small groups (i.e., 5–6 young people). Two members of the research team aided participants when needed. After completing the survey, participants received a $15 Visa gift card and a snack.

Measures. Demographic variables included the age in years, categorized into early adolescence (12–15 years), late adolescence (16–21 years), and emerging adulthood (22–29 years). Other covariates include the youth’s gender (male or female), family living structure (whether living with their parents or alone), and educational status (whether they are graduates, in college, or high school). The youth responded (“yes” or “no”) to whether their parents, siblings, or grandparents had diabetes to determine their family history of diabetes (FH Diabetes). To assess parental substance use, the youth responded to four questions: “Has your father (or mother) ever had problems with consuming too much alcohol,” “Has your father (or mother) ever had problems with using illegal drugs.” (Note: We collected data before modifications in the terminology “illegal drugs.” Thus, hereafter, substance use (SU). Response categories for each SU item were “yes” or “no.” Household hardship, a proxy measure for SES, was assessed using an eight-item scale developed with community members. The scale measured diverse types of household hardships. Example items included: “Do you have problems having trash removed?” “Do you have problems with pests?” Responses were rated on a scale ranging from “not at all” to “all the time.” The scale, which demonstrated acceptable reliability (α = 0.89) with this sample, was categorized to capture youth living in households with no hardship, medium hardship, or severe hardship. Food accessibility was used to measure the availability of sufficient food options and as a proxy for living in a food desert (i.e., where there is no access to healthy foods). A single item, “Is there a large grocery store near you?” with response options “yes” and “no.” Four questions were used to assess the household and youth nutritional habits, asking how often they drank soda and ate fast food, fruits, or vegetables during the past seven days. Responses for fruits and vegetables were categorized into 2 to 4 times a day, a few times a week, or none a week, while soda and fast-food consumption were reverse-coded.

Analysis. StataNow 19.5 was used to conduct analysis. A test of the predictors’ Variance Inflation Factor (VIF) yielded a value of 2.70, indicating that, on average, the predictors were less than moderately correlated (VIFs of less than 5 are generally acceptable). The sample demographic characteristics were highlighted using univariate descriptive statistics. Kendall’s nonparametric bivariate correlation was also used to examine the associations between the variables. Bivariate tests of association were used to examine relationships between a family history of diabetes, demographics, and family and neighborhood risks. A sequential logistic regression was used to explore factors influencing the likelihood of having a family history of diabetes. The first model included youth demographic variables. The second model comprised demographics, nutritional habits, and neighborhood risk factors. The third model included demographics, nutrition, neighborhood, and family risk factors.

Statistical power was examined a priori, using G*Power version 3.1 [40] to ensure an adequate sample size for the proposed analyses, considering an estimated 9,600 youth under 24 who lived in public housing in the study area (Baltimore) in 2022 [41]. The final sample (n = 190) was determined to be ideal, based on a power analysis targeting a medium effect size (1 − β = 0.80, α = 0.05, to limit the risk of Type 1 error) and a power of 80%, which helps reduce the risk of a Type 2 error. Based on the total number of independent variables (14), the expected effect size (0.15), the *p*-value (0.05), and the statistical analysis selected (Linear Multiple Regression: Fixed model, R^2^ increase), this study (n = 190) is adequately powered at 0.91.

## 3. Results

Sample Characteristics. Youth (n = 190, mean age of 18.5, SD, 4.04), included females, 58.42%. Seventy-seven percent (76.84%) of the youth lived with their parents. About thirty-five (35.30%) percent reported having a family history of diabetes. Fifty-four percent of respondents indicated no hardship, 29.0% were experiencing medium hardships, and 16.8% were experiencing severe or above-average household hardships. In terms of paternal alcohol and illegal substance use, 21.6% of the sample reported having a father who had ever had an alcohol problem, and a substance use problem (29.5%). Further, 18.4% percent of the participants reported a mother who had ever had an alcohol problem and a substance use problem (21.1%). Additionally, 20% of the respondents reported limited food accessibility.

*Bivariate Comparisons.* Bivariate tests of association (see Table 1) suggested that the difference between youth who reported frequent soda consumption, household hardship, and parental alcohol and substance use problems was statistically significant by family history of diabetes. Specifically, participants with a family history of diabetes were significantly more likely to report that their mothers had ever had an alcohol (χ^2^ = 4.9 *) or a substance use problem (χ^2^ = 13.6 ***). Furthermore, participants with a family history of diabetes were significantly more likely to report that their fathers had ever had a substance use problem (χ^2^ = 5.8 *). There was a moderate, statistically significant difference between participants with a family history of diabetes who also reported significantly higher levels of household hardships (χ^2^ = 7.5 *). Having a family history of diabetes does not differ by gender, age, educational status, household structure, or food accessibility.

Nonparametric Correlations. Results from Kendall’s nonparametric bivariate correlation (see Table 2) indicated that having a family history of diabetes was significantly and positively correlated to having a mother who ever had substance use problems (r = 0.27 **) and alcohol problems (r = 0.16 *) and a father who had ever had substance use problems (r = 0.17 *) problems. Having a father who has ever had an alcohol use problem was a negligent and insignificant correlation. Furthermore, not eating fruits at least once a week was negatively correlated with a history of diabetes (r = 0.17 *).

*Predictors of Family History of Diabetes*. Results from the sequential logistic regression model were significant [χ^2^ = 52.59 (8); *p* < 0.001] and correctly classified 77.7% of the cases. We also highlight the 95% confidence level (CI) at the z-critical value of about 1.96.

Results from Model 1 indicated that gender and living with parents were unrelated to having a family history of diabetes. Increasing age and youth educational level (being in college) was significantly associated with increased the odds of reporting a family history of diabetes, compared with youth in early adolescence or high school, with the entire CI above one. Results from Model 2, which added food habits, food accessibility, and household hardships, were significant [χ^2^ = 12.63 (2); *p* < 0.05] and indicate that food accessibility was significantly associated with a higher likelihood of having a family history of diabetes (OR = 3.54, *p* < 0.01). Furthermore, consuming fewer or no vegetables in a week compared to those who consumed them daily was significantly associated with higher odds of a family history of diabetes (OR = 8.75 *** and 7.20 **, respectively). However, the large confidence interval suggests a spurious relationship. Being above the sample mean for household hardships (severe hardship) was associated with four times higher odds of having a family history of diabetes than those who reported no hardships (OR = 4.26, *p* < 0.01), with a confidence interval entirely above one (1) and a statistically significant *p*-value. In addition, drinking soda a few times a week was associated with about 80% higher odds of reporting a family history of diabetes, including 84% for those who drank soda 2–4 times daily.

Results from Model 3, which included parental substance use, showed a significant overall fit to the data [χ^2^ = 39.97(2); *p* < 0.001]. Results followed similar patterns as models 1 and 2 and suggested that youth who had a mother who had ever had substance use problems were 6.81 times more likely to report having a family history of diabetes (OR = 6.8 *, *p* < 0.01). Maternal alcohol problems, paternal substance use problems, and paternal alcohol problems were not associated with youth report of a family history of diabetes. The confidence interval suggest a statistically significant association (confidence interval does not include 1). Further, the inclusion of the mother or father ever having alcohol problems and the mother or father ever having a problem with illegal substance use in the model increased the predictive value of the model (pseudo-R, 0.31) and odds of reporting a family history of diabetes (see Table 3).

Results from the Hosmer-Lemeshow goodness-of-fit tests showed that models 1 and 2, with their Χ^2^ (8) statistics of 23.43 ** and 25.85 **, had low *p*-values, suggesting that the predicted outcomes differ significantly from the actual outcomes. Thus, these models may be mis-specified, leading to a rejection of the null hypothesis that the model fits the data adequately. Model 3 (which included parental variables) had a chi2 (8) of 11.19 with a *p*-value greater than 0.05; thus, we fail to reject the null hypothesis that the model fits the data well. In addition, Model 3’s increased pseudo-R suggests that its predicted probabilities align well with the actual outcomes across the sample.

A further likelihood ratio comparison of model 1 and the more complex model 2 yielded an LR Χ^2^ of 37.65, *p* < 0.001, which was highly statistically significant, highlighting that the additional predictors in model 2 supported a better fit to the data (reject the null hypothesis) with improved values in Model 3 (LR Χ^2^ (4) = 24.56 ***).

## 4. Discussion

This study aimed to examine the social determinants, specifically familial and neighborhood risk factors, associated with having a family history of diabetes among African American youth residing in public housing. This exploration is premised on the strong association between a family history of diabetes and children’s health outcomes, which has been indicated in previous research (see, e.g., [16]), along with the constellation of social problems that coalesce in low-SES minority neighborhoods, which may help explicate risk for diabetes. In line with previous research indicating a higher prevalence of diabetes in poor neighborhoods, the results of this study revealed a high prevalence of family history of diabetes among Black youth, 35.3%. This result may be explained by socioeconomic barriers that characterize impoverished neighborhoods [9,17].

The association between household hardships and the likelihood of having a family history of diabetes made in this study has been observed in past research [42] and linked to food insecurity and stress that exacerbate the incidence of and poor management of diabetes. Further, we noted that maternal substance use problems were significantly associated with a higher likelihood of reporting a family history of diabetes. The inclusion of maternal and paternal variables (mother or father ever having problems with alcohol or illegal substance use) in the model increased the odds of reporting a family history of diabetes. This observation is noteworthy and may be linked to elevated levels of stress reported by low-income individuals, which often translates into an increase in diabetes risk in people who are predisposed to the disease. This critical finding can also be explained by maternal caregivers, who often are the primary conduit through which food is made available to the household. Maternal substance use problems may amplify risks of access to nutritionally adequate and safe foods. Similarly, maternal substance use problems may impair a mother’s decision-making abilities, including those related to shopping, cooking, and self-care, which affect well-being.

Food choice behaviors, such as consuming fewer vegetables and fruits or more soda and fast foods, were related to reporting a family history of diabetes. The food choices, specifically less vegetable consumption, negatively and significantly correlated to the availability of large grocery stores, including the consumption of more soda (not significant); this association may seem counterintuitive and challenges the dominant food accessibility discourse that has focused mainly on ‘what is missing’—big grocery stores in poor neighborhoods [24,25]. While the focus on the lack of big grocery stores exposed access-related challenges and how these impinge on the health of individuals in impoverished communities, there may be challenges with capturing what may be more critical to the health of individuals in poor neighborhoods: namely, ‘what is present’—the content of the corner stores located in poor communities.

*Implications.* This study’s findings shift our conceptualization of nutrient access beyond food deserts, highlighting the need to explore the role of food swamps in influencing the health outcomes of low-income individuals and households in impoverished communities. While the availability of a nearby grocery store was associated with a family history of diabetes, this association emphasizes that physical proximity to food sources does not necessarily contribute to access to healthy or affordable nutrition. Food swamps include large and small grocery stores often stocked with outdated/expired produce, highly processed and refined meats, white rice, pasta, and bread. Moving the focus of research from food deserts to food swamps forces us to explore not simply by highlighting the presence of grocery stores but also the quality of food available in them. To advance understanding in this area, future research should employ more comprehensive measures of food access that go beyond geographic proximity, such as Geographic Information Systems (GIS), to help understand poor communities’ challenges in accessing healthy foods.

*Study Limitations*. Results from this study may not be generalizable to all public housing in the U.S., given this location’s unique lower socioeconomic status. Furthermore, our study relied on participants’ reports of a family history of diabetes, and their responses may be limited by family communication, recall, and/or social desirability bias. There is also evidence suggesting that young people have a low level of FHH awareness that may limit the accuracy of their responses [16]. We did not gather data on the grocery stores (store size, content, or specific distance to participant residents), which limited our hypothesis about food deserts. Moreover, survey questions were developed with CAB members, such that measures (e.g., household hardship) may lack psychometric rigor. However, utilizing questions identified by community partners is consistent with the principles of CBPR.

## 5. Conclusions

The study contributes to the body of research examining social determinants associated with a family history of diabetes among African American youth living in public housing. Our study found that familial factors (i.e., household hardship and the mother’s substance use problem) were linked to the likelihood of reporting a family history of diabetes. A constellation of familial and neighborhood factors may be linked to food purchasing capacity and choices. Such findings underscore the need for economic empowerment interventions, including the creation of jobs that pay a living wage. These findings highlight the importance of considering multilevel factors when evaluating diabetes risk in socioeconomically disadvantaged populations. The association between maternal substance use and diabetes history emphasizes the need for gender-sensitive, integrated behavioral healthcare that includes psychosocial interventions in impoverished communities. Overall, this study reinforces the importance of considering contextual factors such as family dynamics and neighborhood conditions in social work and public health practice to address chronic disease risk among youth in impoverished settings.

As highlighted in the role of soda or fast-food consumption, the problem in the neighborhood providing data for this study was not food being inaccessible per se; it may have been food swamps, which are the absence of healthier and more affordable nutritious meals within easy access. Our findings also highlight the need to reassess the measures used to understand access to food in poor neighborhoods and develop more comprehensive measures, capturing what is stocked in the grocery stores and their affordability. Maternal substance use was significantly correlated with having a family history of diabetes, calling attention to the need to address substance use problems in tandem with diabetes prevention programs. This study contributes to the literature addressing the challenges faced by impoverished neighborhoods stemming from poverty and substance use. Although beyond the scope of this study, our results suggest the need to explore familial, household, and neighborhood characteristics and their intergenerational impact on health, particularly among youth in low-resource communities, and to guide preventive care and related interventions.

## Figures and Tables

**Table 1:** Univariate Descriptors and Bivariate Comparisons: Criterion—Family History of Diabetes, n = 190.

		Observations (n) by Family History of Diabetes		
Study Variables	N (%)	No Family History	Has Family History	Χ^2^
Diabetes history		123 (64.7)	67 (35.3)	
Female	111 (58.4)	69	42	0.8
Males	79 (41.6)	54	25	
Age group in years				
Early adolescence (12–15 years)	52 (27.4)	40	12	4.9
Late adolescence (16 to 20 years)	77 (40.5)	45	32	
Emerging adulthood (21 to 29 years)	61 (32.1)	38	23	
Living with parents				
No	44 (23.2)	30	14	0.3
Yes	146 (76.8)	93	53	
Educational status				
Graduate	59 (31.1)	43	16	4.0
In college	15 (7.9)	7	8	
In high school	116 (61.1	73	43	
Household socioeconomic/neighborhood				
Large grocery stores (yes)	152 (80.0)	99	14	0.1
Large grocery stores (no)	38 (20.0)	24	53	
Household Hardship				
No hardship	103 (54.2)	72	31	7.5 *
Medium hardship	55 (29.0)	37	18	
Severe hardship	32 (16.8)	14	18	
Fast food consumption (2–4 times a day)	52 (27.4)	29	23	4.1
A few times a week	43 (22.6)	26	17	
None a week	95 (50)	68	27	
Soda consumption (2–4 times a day)	65 (34.2)	51	14	11.3 **
A few times a week	56 (29.5)	37	19	
None a week	69 (36.3)	35	34	
Fruit consumption (2–4 times a day)	44 (23.2)	25	19	7.2 *
A few times a week	42 (22.1)	22	20	
None a week	104 (54.7)	76	18	
Vegetable consumption (2–4 times a day)	33 (17.4)	23	10	3.1
A few times a week	64 (33.7)	36	28	
None a week	93 (49.0)	64	29	
Mother substance use problem (yes)	40 (21.1)	16	24	13.6 ***
(no)	150 (79.0)	107	43	
Mother’s alcohol problem (yes)	35 (18.4)	17	18	4.9 *
(no)	155 (81.6)	106	49	
Father’s substance use problem (yes)	56 (29.5)	24	27	5.8 *
(no)	134 (70.1)	94	40	
Father’s alcohol problem (yes)	41 (21.6)	23	18	1.7
(no)	149 (78.4)	100	49	

Note: * *p* < 0.05, ** *p* < 0.01, *** *p* < 0.0001.

**Table 2:** Kendall’s nonparametric bivariate correlation (tau-b), n = 190.

Variables	1	2	3	4	5	6	7	8	9	10	11	12	13	14	15
1 Diabetes	1.00														
2 Gender	−0.06	1.00													
3 Age	0.10	−0.26 **	1.00												
4 Live Parent	0.04	0.24 **	−0.54 ***	1.00											
5 Education	0.07	0.05	−0.53 ***	0.23 **	1.00										
6 Grocery	−0.02	0.02	0.06	−0.03	0.02	1.00									
7 Hardship	0.15	−0.07	0.05	−0.08	0.03	0.00	1.00								
8 Fast foods	0.14	0.00	0.20 **	0.03	−0.09	−0.12	0.11	1.00							
9 Soda	−0.23	0.01	0.09	−0.21 **	−0.12	0.02	−0.08	−0.46 ***	1.00						
10 Fruits	−0.17 *	0.16 *	0.02	−0.12 *	−0.08	0.02	−0.05	−0.16 *	0.23 **	1.00					
11 Veggies	−0.05	0.08	−0.07	−0.02	−0.08	−0.13	−0.13 *	−0.10	0.23 **	0.48 ***	1.00				
12 Mom SU	0.27 **	0.17 *	0.29 ***	−0.24 **	−0.15 *	0.03	0.04	0.10	0.08	−0.06	0.10	1.00			
13 Mom Alc	0.16 *	0.26 **	0.02	0.04	−0.02	0.17 *	0.03	−0.11	0.19 **	0.01	0.02	0.48 ***	1.00		
14 Dad SU	0.17 *	−0.05	0.25 **	−0.14 *	−0.14	−0.05	0.08	0.18 **	−0.02	−0.22	−0.17 *	0.40 ***	0.32 ***	1.00	
15 Dad Alc	0.09	0.10	−0.06	−0.08	0.09	−0.03	0.04	−0.16 *	−0.03	−0.03	−0.08	0.14	0.38 ***	0.36 ***	1.00

Note: * *p* < 0.05, ** *p* < 0.01, *** *p* < 0.0001; Alc: Alcohol, SU: Substance use.

**Table 3:** Hierarchical Logistic Regression Model: Criterion—Family History of Diabetes (n = 190).

	Model 1	Model 2	Model 3
Variables	OR [95% CI]	OR [95% CI]	OR [95% CI]
Constant	0.00 *** [0.01, 0.25]	0.01 *** [0.00, 0.14]	0.04 *** [0.00, 0.09]
Gender: Male	0.85 [0.44, 1.66]	1.06 [0.48, 2.33]	0.44 [0.17, 1.16]
Age: Late adolescence, 16–20 years	3.00 ** [1.31, 6.84]	3.88 * [1.36, 11.04]	3.33 * [1.06, 10.40]
Emerging adult, 21–29 years	6.01 ** [1.65, 21.95]	11.72 ** [2.14, 64.07]	6.50 * [1.04, 40.53]
Living with parents (yes)	2.55 [0.94, 6.89]	2.44 [0.73, 8.22]	3.07 [0.80, 11.68]
Education: In college	3.45 * [1.03, 11.51]	5.30 * [1.10, 25.44]	5.97 * [1.13, 31.50]
In high school	3.00 * [1.20, 7.48]	4.68 ** [1.60, 13.73]	6.60 * [1.81, 24.10]
Large grocery stores (yes)		1.55 [0.57, 4.21]	1.28 [0.42, 3.86]
Fast food consumption			
A few times a week		1.19 [0.43, 3.24]	0.81 [0.26, 2.50]
2 to 4 times a day		0.79 [0.24, 2.61]	0.81 [0.20, 3.28]
Soda consumption			
A few times a week		0.20 ** 0.06, 0.61]	0.12 ** [0.31, 0.47]
2 to 4 times a day		0.16 ** [0.05, 0.51]	0.06 *** [0.01, 0.26]
Fruit consumption			
A few times a week		2.35 [0.71, 7.76]	5.90 * [1.39, 25,04]
None in a week		0.36 [0.13, 1.04]	0.79 [0.23, 2.67]
Vegetable consumption			
A few times a week		8.75 *** [2.64, 28.97]	15.08 *** [3.64, 62.45]
None in a week		7.20 ** [1.92, 27.04]	7.72 ** [1.73, 34.48]
Hardship. Medium hardship		1.37 [0.56, 3.37]	1.76 [0.61, 5.12]
Severe hardship		4.26 ** [1.54, 11.77]	5.82 ** [1.84, 18.41]
Mother substance use problem (yes)			6.81 * [1.58, 29.31]
Mother’s alcohol problem (yes)			3.67 [0.90, 15.06]
Father’s substance use problem (yes)			1.33 [0.44, 3.99]
Father’s alcohol problem (yes)			0.53 [0.18, 1.76]
Pseudo R^2^	0.06	0.21	0.31
Hosmer–Lemeshow Χ^2^	27.43 **	25.85 **	11.19
Intercept Predictive Margin	35.3% *** [28.7%, 41.8%]	35.3% *** [29.4%, 41.1%]	35.3% *** [29.8%, 40.7%]
2 × 2 Contingency	1.70 [0.68, 4.13]	1.70 [0.68, 4.13]	1.70 [0.68, 4.13]
Χ^2^	1.68	1.68	168

Note: * *p* < 0.05, ** *p* < 0.01, *** *p* < 0.0001.

## Data Availability

The original contributions presented in this study are included in the article. Further inquiries can be directed to the corresponding author.

## Abbreviations

The following abbreviations are used in this manuscript:

ACE	Adverse Childhood Experiences
CAB	Community Advisory Board
GIS	Geographic Information System
FHH	Family Health History
CDC	Centers for Disease Control
RDS	Respondent-Driven Sampling
OR	Odds Ratio
SDoH	Social Determinants of Health
SES	Socioeconomic Status
